# Characteristics of Colonic Pseudo-Obstruction Post Ileostomy Reversal: A Retrospective Study and Scoping Review of the Literature

**DOI:** 10.7759/cureus.99078

**Published:** 2025-12-12

**Authors:** Zirong Yu, Ferdinand Ong, Timothy Slack

**Affiliations:** 1 General Surgery, Princess Alexandra Hospital, Woolloongabba, AUS

**Keywords:** acute colonic pseudo-obstruction, colonic pseudo-obstruction, general surgery and colorectal surgery, ileostomy closure, ileostomy reversal, lower gastrointestinal or colorectal surgery

## Abstract

Colonic pseudo-obstruction (CPO) is characterized by dilatation of the colon without an anatomical or mechanical obstruction. There are multiple etiologies that may lead to this phenomenon, including post-major abdominal or pelvic surgery, critical illness states, drug-induced effects, and severe electrolyte imbalances, to name a few. An ileostomy closure is a commonly performed surgical procedure that restores the continuity of the gastrointestinal tract post-significant previous colorectal resection. Common complications have been known to occur, such as small bowel ileus, anastomotic leak, wound site infections; however, reports of CPO post-ileostomy closure have not been well reported or studied. This study aimed to evaluate the characteristics, management, and outcomes of patients treated for CPO after loop ileostomy closure and to identify the existing evidence in the literature on this uncommon phenomenon.

We conducted a retrospective study of all patients from Princess Alexandra Hospital who developed CPO after ileostomy closure in the past 10 years. Electronic records were accessed to find patient demographic and clinical details, including primary surgery, whether or not radiation was applied, co-morbidities, as well as hospital length of stay and post-operative outcomes. Subsequently, a scoping review was conducted of literature to identify all studies reporting CPO after ileostomy closure. Comparisons were made between our results and those of the scoping review. Nine patients were included in our retrospective study. Seven patients required ileostomy after ultra-low anterior resection (ULAR) for cancer, and two patients for perforated diverticulitis. Mean time to reversal was 7.78 months. Three patients had neoadjuvant and adjuvant chemotherapy, and three patients had neoadjuvant therapy only. CPO diagnosis was made on radiological imaging, and the mean length of hospital stay for all patients was 9.5 days. Mean CPO resolution time was 4.8 days. Scoping review identified 200 studies; however, only one study qualified for inclusion. Five patients were included who underwent ULAR with ileostomy, with four patients requiring neoadjuvant and adjuvant therapy. Mean time to reversal was 8.3 months. Both the retrospective study and scoping review found similar patient characteristics in those who developed CPO post-ileostomy closure. In particular, it would appear that in an irradiated pelvis, the risk of CPO development appeared higher; however, larger patient studies are required to inform better statistical analyses and draw more correlations.

## Introduction

Acute colonic pseudo-obstruction (CPO), also known as Ogilvie’s syndrome, is a form of paralytic ileus that affects the large bowel and is characterized by dilatation of the bowels in the absence of an identifiable anatomical obstruction [1]. This condition was first described by William Ogilvie in the 20th century, and to this day, mortality can be expected to exceed 15% in uncomplicated cases and 30-40% in complicated disease [2,3]. Most cases of CPO are found in patients who have undergone major abdominal surgery, the elderly, or those who are critically ill, and present with symptoms of bowel obstruction, including nausea, vomiting, abdominal distension, and obstipation [1,3]. The pathophysiology is unclear, with no definitive causes currently found; however, many clinical conditions have been associated, such as cerebrovascular accidents, trauma, surgery, metabolic imbalances, cardiac disease, medications, and immobility, to name a few [3,4]. Restoration of gastrointestinal continuity after ileostomy closure is a common and usually a technically straightforward surgical procedure. Mechanical bowel obstruction remains one of the more commonly reported post-operative complications [5]. Considering CPO as a differential post-loop-ileostomy closure is essential, as any delay to diagnosis in these comorbid patients with recent surgery and prolonged de-functioned gut activity can have significant morbidity [6-8].

Hence, this study aimed to evaluate the characteristics, management, and outcomes for patients treated for CPO after ileostomy closure at our institution and to identify the currently existing evidence within the literature on this uncommon but very specific phenomenon. In doing so, we seek to bring more information and awareness to this easily misdiagnosed condition, but also identify the impact of delayed diagnosis and aim to provide optimal suggestions for the management of this uncommon complication.

## Materials and methods

Case series

A retrospective study was undertaken at Princess Alexandra Hospital, a major tertiary trauma center located in Brisbane, Queensland, Australia. Data were collated from the electronic medical database within hospital records for all patients aged 18 years and over who were diagnosed with colonic pseudo-obstruction following ileostomy closure from January 2013 through July 2023. Bowel obstruction due to other causes, such as mechanical, isolated small bowel ileus, or non-specific bowel obstruction, was excluded. Patients who did not undergo ileostomy reversal were also excluded. Patients who did not have radiological or endoscopic confirmation of CPO were also excluded.

Baseline patient characteristics were collected, including age, sex, gender, comorbidities, functional status, peri-operative details, and rationale for ileostomy. Comorbid burden for patients was gathered by using the Charlson Comorbidity Index (CCI), a validated method of classifying prognostic comorbidity [9]. The CCI assigns weighted scores to specific medical conditions, which are totaled to produce a composite comorbidity score predictive of mortality risk. Individual comorbidities were identified from patients’ clinical records and coded diagnoses. The total Charlson score was calculated according to the original 1987 definitions, without reproducing the proprietary weighting table [9]. The Eastern Cooperative Oncology Group (ECOG) scores were also gathered from patient data and records [10]. Further clinical data, including biochemical analyses, baseline laboratory results, radiological findings, operative reports, symptomatology, and examination findings, were also gathered. The post-operative recovery, length of stay, and complications were recorded. Ethics approval from the Metro South Health Human Research Ethics Committee was obtained prior to the commencement of the study.

Scoping review

We performed a scoping review of the literature following the guidelines and framework set by the Joanna Briggs Institute Scoping Review Methodology [11]. A review team was formed comprising two individual surgical doctors (ZY, FO). A key feature of our study methodology was the combination of previous experience in literature reviews alongside senior surgical subject matter expertise in colonic diseases. A scoping review methodology was chosen as it was the most appropriate methodology for elucidating a particular research question that has yet to be comprehensively studied, investigated, and reviewed [12]. Additionally, given the specificity of our research question and the likely paucity of evidence available, a scoping review provided the ability to identify the available information present in the literature as well as gaps in the knowledge, while identifying key characteristics or factors related to our study [13].

Inclusion criteria and study types

All inclusion and exclusion criteria for our search were the same as those used in our retrospective case series, except for the timeline restriction, as we identified all studies in the English literature on patients aged 18 years and older diagnosed with CPO post-ileostomy closure. Given the very specific parameters of our research question, as well as the rarity of the phenomenon, all published data were included, such as case reports and other case series, with no restrictions placed on time.

Search terms and strategy

In our strategy, an initial search of PubMed was conducted with generic terms (colonic pseudo-obstruction, ileostomy, ileostomy closure, obstruction) to identify general text words contained in the title and abstract, as well as any further terms that can be used as alternate search terms. A preliminary scope of the literature available was also noted at this stage, and relevant articles were considered. Following on, a second, more detailed search was undertaken using the following specific search terms and Medical Subject Headings (MeSH) terms across MEDLINE, Embase, and CINAHL databases. These included "MH Colonic Pseudo-Obstruction OR Colonic Pseudo Obstruction OR Pseudo-Obstruction OR Pseudo Obstruction OR MH Intestinal Pseudo-Obstruction OR Intestinal pseudo obstruction OR Ogilvie Syndrome) AND (MH Ileostomy OR Ileostomy N3 OR reversal OR closure." The reference list of all identified studies was also searched for additional studies that met the inclusion criteria. As a final step, a generic search of ResearchGate was used to identify any additional literature (grey literature) that may not have been readily available in more conventional databases.

Study selection and data extraction

The selection of studies was performed by two independent authors (ZY, FO) over two stages. Initial screening of title and abstract was reviewed and then full text review against the inclusion criteria. Duplicates were removed, and any disagreements were discussed and resolved by all authors.

## Results

A total of nine patients met our inclusion criteria within the designated search period. Median age at diagnosis was 70.4 years, and all patients were male. Baseline characteristics, comorbidities, clinical data, management, and outcomes are outlined in Table 1. Of note, one patient received adjuvant chemotherapy post-index operation for rectal cancer, and one other patient was immunocompromised on a background of renal transplantation.

**Table 1:** Patient characteristics - ileostomy reversal series. CCI: Charlson Comorbidity Index; ECOG: Eastern Cooperative Oncology Group; AXR: abdominal X-ray; ULAR: ultra-low anterior resection; TVA: tubulovillous adenoma; HGD: high-grade dysplasia; IHD: ischemic heart disease; CKD: chronic kidney disease; DVT: deep vein thrombosis; COPD: chronic obstructive pulmonary disease; GORD: gastroesophageal reflux disease; OSA: Obstructive sleep apnea; T2DM: type 2 diabetes mellitus; CABG: coronary artery bypass grafting; HAR: high anterior resection; AF: atrial fibrillation

Age (years)	Sex	CCI	ECOG	Comorbidities	Reason for ileostomy	Ileostomy duration (months)	Radiotherapy/chemotherapy	Pelvic sepsis	Post-operative day of diagnosis	Diagnostic investigation	Duration of obstruction (days)	Management	Length of stay (days)	Complications	Outcome
70	M	2	0	Hypertension, benign prostate hyperplasia	ULAR for rectal cancer	9	Neoadjuvant chemoradiotherapy and adjuvant chemotherapy	No	3	AXR	4	Endoscopic decompression on post-operative day three, gastrograffin	7	No	Discharged home
74	M	4	1	IHD, CKD, dyslipidemia, hypertension	ULAR for TVA with HGD	10	No	Yes, anastomotic leak managed with percutaneous and transrectal drainage	3	CT	9	Endoscopic decompression on post-operative day three, antibiotics for possible colitis	18	No	Discharged home
72	M	3	1	Provoked DVT	ULAR for rectal cancer	13	Neoadjuvant chemoradiotherapy and adjuvant chemotherapy	No	3	AXR	4	Medical therapy. Subsequent operation for necrosis	18	Right colon necrosis requiring laparotomy, right hemicolectomy, and end ileostomy	Discharged home
84	M	5	1	Asthma, COPD, GORD	ULAR for rectal cancer	4	Neoadjuvant radiotherapy	No	3	AXR, CT	5	Endoscopic decompression on post-operative day four, gastrograffin	8	No	Discharged home
65	M	2	0	Hypertension, hypercholesterolemia	ULAR for rectal cancer	13	Neoadjuvant short-course radiotherapy and adjuvant chemotherapy	No	3	AXR, CT	5	Endoscopic decompression on post-operative day five	8	No	Discharged home
67	M	3	1	IHD, hypertension	ULAR for rectal cancer	6	Neoadjuvant chemoradiotherapy and adjuvant chemotherapy	No	3	AXR	4	Medical therapy, antibiotics for hospital-acquired pneumonia, and Salmonella on stool culture	7	Hospital-acquired pneumonia managed with antibiotics	Discharged home
68	M	3	1	T2DM, OSA, GORD, hypertension, arthritis	HAR for perforated diverticulitis	9	No	No	3	AXR, CT	5	Endoscopic decompression on post-operative day seven	9	No	Discharged home
72	M	5	1	IHD (CABG), renal transplant, gastric ulcer, hypertension, shingles	Reversal of Hartmann's procedure following perforated diverticulitis	3	No	No	5	CT	5	Medical therapy, flatus tube, and antibiotics for possible colitis	9	No	Discharged home
62	M	2	0	T2DM, AF, hypertension	ULAR for rectal cancer	3	Neoadjuvant short-course radiotherapy	No	3	AXR, CT	3	Endoscopic decompression on post-operative day four	8	No	Discharged home

Seven patients (78%) required their index loop ileostomy after ultra-low anterior resection (ULAR) for rectal cancer, and two patients (22%) required loop ileostomy for perforated diverticular disease. The mean wait time to ileostomy reversal was 7.78 (range: 3-13) months. The mean time delay for the rectal cancer group was 8.29 months, and for the perforated diverticular disease group, it was six months.

Three patients (33%) had both neoadjuvant chemoradiotherapy (capecitabine with radiation) and adjuvant chemotherapy. Three patients (33%) had neoadjuvant radiotherapy, with only one patient having follow-up adjuvant chemotherapy. One patient had ULAR for a tubular adenoma with high-grade dysplasia and received no neoadjuvant or adjuvant therapy. The remaining two patients had stomal closure post-resection for diverticulitis; hence, no oncological treatment was required.

The predominant symptom on CPO diagnosis was abdominal distension (78%), followed by vomiting (44%). Two patients had obstipation, whereas three had overflow diarrhea. Four patients (44%) had tachycardia, and two patients (22%) showed evidence of worsening respiratory distress. Three patients (33%) were febrile at the time of diagnosis.

All diagnoses of CPO were made on radiological findings showcasing clear evidence of colonic dilatation alongside clinical presentation of obstipation, with three (33%) cases diagnosed solely on having plain abdominal X-ray (AXR), and the remaining six (67%) patients having abdominal CT scans. CPO was diagnosed in eight (89%) patients on day three after ileostomy closure and one patient on day five post closure. All patients underwent initial conservative management of CPO, including bowel rest, decompression, and replacement of electrolyte deficiencies. The mean resolution time for CPO in all patients was 4.8 (range: 3-9) days.

All patients were initially given oral Gastrografin, with only three patients (33%) attaining resolution of the CPO without further intervention. Six patients (67%) proceeded to require endoscopic colonic decompression on failing conservative management on a mean of 4.3 days post-ileostomy closure. Stool cultures were taken on the three patients who presented with overflow diarrhea, with only one sample identifying the presence of *Salmonella enterica*, which was subsequently treated with intravenous antibiotics.

The mean total length of hospital stay for all patients was 9.5 (range: 7-18) days, and there were no readmissions. CCI scores ranged from two to five, with a mean score of 3.2. One patient’s hospital course was complicated by hospital-acquired pneumonia, requiring an extended hospital stay for intravenous antibiotics. Of particular note, one patient who failed non-operative management of CPO required a laparotomy, right hemicolectomy, and end ileostomy for right-sided colonic necrosis.

In totality, six patients underwent endoscopic (interventional) decompression of their bowels, and three patients were managed conservatively with bowel rest, gentle aperients, and optimization of electrolytes. Four patients had concomitant treatment for colitis with intravenous antibiotics. All patients resumed normal bowel habits at the time of discharge.

## Discussion

Our study demonstrates the overall rarity of colonic pseudo-obstruction after ileostomy reversal in the medical and surgical literature. Although uncommonly encountered post-operatively, untreated or poorly managed CPO can contribute to significant patient morbidity, and as such, a thorough appreciation of its causes and associated risk factors is required. Only nine patients were diagnosed and treated for CPO after ileostomy closure at our institution over a 10-year period. Scoping review of the medical literature revealed only one study that satisfied our inclusion criteria, detailing five patients managed for CPO post-ileostomy reversal [14].

All patients in our study were men older than 60 years, a factor also reflected in the findings of the scoping review and study. Most of our patients had several comorbidities with a median CCI of three (range: 2-5). Radiological imaging was used for diagnosis in both our studies and the scoping review, with a median day of diagnosis of day three post-operatively in our study, compared with day five post-operatively in the study reviewed [14]. In our case series, the mean length of stay was 9.5 (range: 7-18) days, compared with 20.3 (range: 12-29) days in the reviewed study; symptoms resolved on average within five days in our patients. Statistical analysis, however, could not be performed in either cohort due to paucity in participant numbers.

In our retrospective study, the majority of patients underwent endoscopic decompression of the bowels, which contrasts with the scoping review study, in which most of their patients had resolution following bedside flatus tube insertion [14]. Management options for CPO range from conservative medical therapy to endoscopic intervention and surgical resection, with the main principle of treatment being decompression of the dilated colon to prevent bowel ischemia and perforation [15,16]. Multiple guidelines within the international literature strongly advocate for endoscopic decompression as the first choice for patients with CPO [17-19], demonstrating successful resolution of obstruction in up to 69% of patients [20]. Flatus tube placement, however, has limited data in the literature, with reports suggesting increased patient discomfort and repeated tube removal and replacements, adding to both clinician and patient frustration [21]. However, due to a lack of prospective controlled studies and extensive research, obtaining high-quality results in this area remains elusive. Nevertheless, the differing management options between our series and the scoping review may reflect in the resultant different total length of stay or duration to resolution of symptoms; however, much larger sample sizes are required to confirm this.

Scoping review

Search Results

A review of the databases, their reference lists, and the grey literature initially identified 200 studies. After extensive reviews of the abstracts and removing duplicates, only one study satisfied the requirements of our inclusion criteria [14]. Studies were excluded due to some of the following reasons: pediatric studies (n=8), narrative reviews with unspecific details (n=5), colostomy reversal (n=1), different operative techniques for ileostomy reversal (n=17), comparing optimal time for ileostomy reversal (n=10) and unspecific complications of post-operative ileus, small bowel obstruction, and studies without clear evidence of colonic obstruction (n=22). The remaining studies (n=136) that were screened were excluded due to a lack of specificity and relevance to our studied topic.

Study Characteristics

The characteristics of our chosen study and selected data outcomes are displayed in Table 2. From one article, five patients met our inclusion criteria [14]. The mean age was 61.8 (range: 49-70) years, and all patients were male. All patients had an ECOG score of 1 with good baseline health and function [10]. All patients required ileostomy after ultra-low anterior resection for rectal cancer, with four patients requiring neoadjuvant chemoradiotherapy and adjuvant chemotherapy for higher-risk rectal cancer. The mean time to ileostomy reversal was 8.3 (range 4-12) months.

**Table 2:** Scoping review results. ULAR: ultra-low anterior resection; ECOG: Eastern Cooperative Oncology Group; ASA: American Society of Anesthesiologists; Ca: cancer

Study	Study type	Location	Sample size	Age in years (mean)	Sex	ASA	ECOG	Comorbidities	Diagnosis	Reason for ileostomy	Ileostomy duration	Management	Outcomes
Tan et al. (2023) [14]	Case Series	Singapore	n=5	54	M	1	1	No comorbidities	Colonic pseudo-obstruction	Robotic ULAR (rectal Ca)	7 months	Medical therapy, multiple attempts with sigmoidoscopic decompression to no success. Finally required defunctioning colostomy.	Permanent defunctioning colostomy
				70	M	2	1	No comorbidities	Colonic pseudo-obstruction	Laparoscopic ULAR (rectal Ca)	4 months	Medical therapy, stool softeners, and multiple attempts at decompression with rectal tube insertions. No resolution of symptoms. Unfortunate perforation of cecum, leading to sepsis and intra-operative cardiac arrest.	Death
				69	M	2	1	No comorbidities	Colonic pseudo-obstruction	Robotic ULAR (rectal Ca)	12 months	Medical therapy, multiple bedside flatus tube insertions. Successful decompression and passing of bowels.	Discharged home
				67	M	1	1	No comorbidities	Colonic pseudo-obstruction	Robotic ULAR (rectal Ca)	10 months	Medical therapy, multiple bedside flatus tube insertions. Successful decompression and passing of bowels.	Discharged home
				49	M	1	1	No comorbidities	Colonic pseudo-obstruction	Robotic ULAR (rectal Ca)	8.5 months	Medical therapy, multiple bedside flatus tube insertions. Successful decompression and passing of bowels.	Discharged home

The diagnosis of colonic pseudo-obstruction was made with a combination of both clinical and radiographic findings, with a mean time to diagnosis of five days post-ileostomy closure. All patients reported intermittent passage of poorly formed stools, which the authors noted added to the diagnostic difficulty.

All patients were initially trialled on medical therapy consisting of stool softeners and prokinetic agents. Three patients had successful resolution of symptoms with insertion of flatus tube at bedside; one patient required creation of a defunctioning colostomy after multiple failed attempts at sigmoidoscopic decompression. This patient’s clinical course was later complicated by a rectoprostatic fistula requiring transanal suture repair. One patient, unfortunately, had a cecal perforation after multiple attempts at rectal tube insertions, which led to sepsis, intra-operative cardiac arrest, and death. This represented an overall mortality of 20% in this series.

The mean length of stay for the patients was 20.3 (range: 12-29) days, and no data were reported for symptomatic resolution time in the study. Given the small sample size and the overall low level of evidence in the included study, no further statistical analysis was performed.

Our study and scoping review sought to investigate potential etiologies and risk factors for CPO, specifically among patients who underwent ileostomy closure. Details on these for CPO in the overall population are certainly robust and well-reported in the medical and surgical literature [4]. Multiple contributors, namely trauma, anesthesia, certain medications, chronic disease, frailty, and malignancy, have been reported as potential precursors to this disease; however, to this date, there has been no clearly established pathophysiology of CPO [1,4,20]. However, a strong consensus remains on the urgent need to alleviate distension post-operatively, as it is well known that colonic obstruction distal to the closed ileostomy site is a notable contributor to increased back pressure and hence the risk of anastomotic leak [22].

An emerging concept of thought is the relationship of patients undergoing radiotherapy or having pelvic sepsis and its potential impact on CPO following ileostomy closure. Within the literature, studies have shown that fibrosis as a result of neoadjuvant radiation exposure can present with neorectal hyposensitivity on ileostomy closure [23], which is corroborated by other authors who suggest that radiotherapy does indeed impair functional outcomes in patients as measured in manometric studies [24,25]. These results are, to a degree, reflected in our patients, most of whom have undergone levels of neoadjuvant radiotherapy prior to ULAR. Pelvic sepsis, as seen in one of our patients, is also a likely compounding factor leading to worsened fibrosis and decreased neorectal compliance [26]. This is certainly reflected well by our reviewed study, which reported four out of five patients with pelvic sepsis in the context of CPO development post their eventual ileostomy closure [14]. Prolonged inactivity of the pelvic floor musculature, in addition to potential neuronal injuries as a result of ULARs, has also been suggested as a possible predeterminant of CPO post-ileostomy closure [26,27]. Due to the inherent delay in closure (to allow time for adjuvant therapy), prolonged colonic disuse and atrophy may also result in colonic dysfunction and CPO. Some studies suggest that earlier closure may be beneficial to avoid abnormal neural regeneration that may predispose patients to colonic dysfunction and obstruction [28].

Although our scoping review did not generate extensive evidence to formulate a causative relationship between CPO and ileostomy closures, it did reveal the existence of this phenomenon in other institutions worldwide and, more importantly, highlighted a noticeable gap within the surgical literature on this rare but important disease [14]. While none of the scoping reviews and examined series provided grounds for further statistical or meta-analyses to identify relationships between common patient comorbidities and risk factors for the development of CPO post-ileostomy closure, it certainly garners evidence to support the need to undertake further research in this area. As seen in both our study and the review, prolonged hospitalization, increased risk of post-operative complications, and patient mortality and morbidity can be associated with CPO post-ileostomy reversal, thus demonstrating the potential diverse impacts this condition may impose on patient outcomes [29,30]. Perhaps the most effective way to apply this new information is to raise awareness within the surgical community of the potential development of CPO after ileostomy closure, even in the misleading context of a patient who continues to pass bowel motions after ileostomy closure [14].

The primary limitation of our scoping review is the poor quality and limited number of studies present for further statistical analyses. However, the goal of a scoping review is to identify research that has been conducted and to identify gaps in the literature in preparation for future research, not necessarily to draw conclusions or to assess quality. While quality assessment and conclusive evidence were not the goal of our review, these factors should be considered in future research and in designing systematic reviews that can better target a particular clinical question.

## Conclusions

In summary, our retrospective study, although small in cohort size, provides an initial insight into the potential relationship between ileostomy closure and the development of CPO. One emerging theory has concentrated on exposure to neoadjuvant therapy impairing subsequent functional outcomes, potentially contributing to colonic dysfunction on closure. This was seen in all patients in our study and is corroborated by our identified study from the scoping review; however, larger patient cohorts will be required before statistical analyses and causative relationships can be drawn. The scoping review itself provided essential information on the gaps within the surgical literature where more prospective and systematic research is required to better address this rare and specific phenomenon.
